# Investigating the addition of oral HIV self-tests among populations with high testing coverage – Do they add value? Lessons from a study in Khayelitsha, South Africa

**DOI:** 10.1371/journal.pone.0215454

**Published:** 2019-05-02

**Authors:** Hazel Ann Moore, Carol A. Metcalf, Tali Cassidy, Damian Hacking, Amir Shroufi, Sarah Jane Steele, Laura Trivino Duran, Tom Ellman

**Affiliations:** 1 Médecins Sans Frontières, Khayelitsha Project, Cape Town, South Africa; 2 Division of Public Health Medicine, School of Public Health and Family Medicine, University of Cape Town, Cape Town, South Africa; 3 Médecins Sans Frontières, Cape Town, South Africa; 4 Southern Africa Medical Unit, Médecins Sans Frontières, Cape Town, South Africa; University of KwaZulu-Natal, SOUTH AFRICA

## Abstract

**Introduction:**

HIV self-testing (HIVST) offers a useful addition to HIV testing services and enables individuals to test privately. Despite recommendations to the contrary, repeat HIV testing is frequent among people already on anti-retroviral treatment (ART) and there are concerns that oral self-testing might lead to false negative results. A study was conducted in Khayelitsha, South Africa, to assess feasibility and uptake of HIVST and linkage-to-care following HIVST.

**Methods:**

Participants were recruited at two health facilities from 1 March 2016 to 31 March 2017. People under 18 years, or with self-reported previously-diagnosed HIV infection, were excluded. Participants received an OraQuick Rapid HIV-1/2 Antibody kit, and reported their HIVST results by pre-paid text message (SMS) or by returning to the facility. Those not reporting within 7 days were contacted by phone. Electronic and paper-based clinical and laboratory records were retrospectively examined for all participants to identify known HIV outcomes, after matching for name, date of birth, and sex. These findings were compared with self-reported HIVST results where available.

**Results:**

Of 639 participants, 401 (62.8%) self-reported a negative HIVST result, 27 (4.2%) a positive result, and 211 (33.0%) did not report. The record search identified that of the 401 participants self-reporting a negative HIVST result, 19 (4.7%) were already known to be HIV positive; of the 27 self-reporting positive, 12 (44%) were known HIV positive. Overall, records showed 57/639 (8.9%) were HIV positive of whom 39/57 (68.4%) had previously-diagnosed infection and 18/57 (31.6%) newly-diagnosed infection. Of the 428 participants who self-reported a result, 366 (85.5%) reported by SMS.

**Conclusions:**

HIVST can improve HIV testing uptake and linkage to care. SMS is acceptable for reporting HIVST results but negative self-reports by participants may be unreliable. Use of HIVST by individuals on ART is frequent despite recommendations to the contrary and its implications need further consideration.

## Introduction

The United Nations AIDS Program (UNAIDS) 90-90-90 target aims to ensure that 90% of people living with HIV (PLHIV) will know their status by 2020 [1]. Worldwide, 25% of PLHIV do not know their status [2], and in South Africa this figure is 15% [3]. HIV incidence in South Africa has decreased (from 1.72% annually in 2012 to 0.79% annually in 2017), but at the end of 2016 only 70.6% of the 7.9 million people with HIV infection were on antiretroviral therapy (ART) [3,4]. HIV self-testing (HIVST) has been shown to increase uptake and frequency of HIV testing [5,6] and has the potential to provide access to testing for high-risk, untested, hard-to-reach and test averse populations [7]. The World Health Organisation (WHO) recommends HIVST as an additional approach to HIV testing services [5] and WHO prequalified OraQuick HIV Self-Test (OraSure Technologies Inc) in July 2017 [8] and a blood-based HIV self-test in 2018 [9].

HIVST is defined as “the process whereby an individual collects their own specimen (blood or oral fluid), performs HIV testing using a rapid diagnostic test and interprets the result themselves either assisted or unassisted” [5]. By July 2018, 59 countries had adopted HIVST policies, with many additional countries currently preparing to introduce HIVST [10]. In South Africa, the HIV Clinicians’ Society developed HIVST guidelines [11] and a national guideline on self-testing (referred to as self-screening in these guidelines because of the need for confirmatory testing) was published in May 2018 [12].

With the decrease in cost in 2017 of HIVST tests from US$ 7.50–15.00 to US$2.00 across 50 high burden and certain lower- and middle\-income countries, including South Africa, Zimbabwe, Uganda, Kenya and Swaziland, HIVST has become more affordable [13,14]. Oral fluid has a lower HIV antibody concentration than blood, and this results in a slightly higher false-negative rate than blood-based tests [15]. Positive HIVST results require confirmatory testing in accordance with national testing guidelines [12]. HIVST, used as a screening test, may reduce barriers to access for people wanting to test for HIV, and decrease burden on health care providers [5,16]. Only those individuals with positive HIVST results need to attend health services for confirmatory testing and treatment, and those screening negative can self-test at regular intervals and be directed to preventive services (including male circumcision and PrEP), resulting in in better use of limited resources [5].

Repeat testing of those with a negative status is encouraged, especially among individuals with a high risk of HIV exposure, and those who may be in the window period. Some PLHIV—have been found to re-test repeatedly, despite health authorities discouraging repeat testing for those on ART (because of the risk of false-negative results) [12]. Because the HIV test measures antibodies and not the virus, people on ART who have viral suppression, and those who start ART very soon after acquiring HIV infection may have very low, or attenuated, HIV antibodies, leading to false negative results [17–19].

With careful instruction, HIVST can be performed accurately by the majority of self-testers [20,21]. Some studies have raised concerns about the possibility of coercion of partners [22] and poor linkage to care [6,23].

Research is on-going to identify the best distribution strategies to facilitate HIVST use. Community-based and partner-delivered strategies have been most widely researched [24–27]. A formative study carried-out in Khayelitsha in preparation for this study, found that HIVST was well accepted, and had a potential to increase access to HIV testing, especially among men and youth [28], which have been identified as hard-to-reach groups by several studies [27,29,30].

The primary objectives of the study were to assess whether the provision of unsupervised HIVST was feasible and increased uptake of routine HTS, particularly in those reluctant to use clinic-based testing and to assess post-study linkage to care. The study investigated the use of an SMS for participants to report their results to the study as well as a means for reminding participants of the need for confirmatory testing. A secondary objective, was to investigate the use of self-tests by those already aware of their positive status (based on matched clinic records) and its impact on their ART engagement.

## Methods

### Ethical statement

Ethics approval was obtained from the University of Cape Town Human Research Ethics Committee (HREC 567/2014) and the MSF Ethics Review Board (Protocol 1432), and permission to conduct the study in the two health facilities was granted by the Western Cape Department of Health. All participants in the study provided written informed consent.

### Design and study setting

The study had a cohort design. Participants were recruited at two health facilities in Khayelitsha, using convenience sampling. Khayelitsha is a peri-urban area near Cape Town. It has a population of approximately 500,000 people, with 60% of inhabitants living in informal housing. Khayelitsha has a high HIV prevalence (34% prevalence among pregnant women in 2015) [31] and a high HIV testing coverage [32]. Literacy level is over 90% [33].

Two testing sites were used. Facility 1 is a community health centre (CHC) that provides comprehensive primary health care services, including acute and chronic disease care, reproductive health (including deliveries), HIV and tuberculosis diagnosis and care. As with other community health centres in the area, the majority of patients are female with approximately 60% of adults testing for HIV (excluding antenatal visits) being female.

Facility 2 is a wellness hub that provides basic screening services for common conditions including tuberculosis, sexually-transmitted infections, pregnancy, hypertension, diabetes, and provides family planning services. All patients are seen first by a counsellor, who routinely offers provider-initiated counselling and testing (PICT), using rapid HIV tests. Patients screening positive for any health condition are referred to local health facilities for further management. Approximately 98% of users are female.

### Recruitment

Participant recruitment was done from 1 March 2016 to 31 March 2017. Participants were required to be at least 18 years of age due to legal requirements for parental consent among minors participating in research. People with confirmed HIV infection, based on self-report, were ineligible to participate. Participants were required to provide a telephone contact number and consent to telephonic follow-up for study purposes. Study staff publicised the availability of HIVST in public waiting areas of the clinics, but screening and all study–related activities were conducted in a private room to maintain confidentiality. Study staff and clinic staff distributed flyers publicising the study in the clinics and in the community and study participants received flyers to distribute to partners and other associates potentially interested in taking part in the study.

Pre-existing patient flow was different at the two study sites and in order to allow normal practises to be maintained, recruitment strategies varied slightly across sites. At Facility 1, a study counsellor gave HIV health education talks in general waiting areas and invited patients interested in HIVST to be screened in a private room.

At Facility 2, where all patients were routinely seen by a clinic counsellor and offered PICT, only those who declined PICT or asked specifically for HIVST were invited to enrol in the study.

### Enrolment

Each participant provided written informed consent. As part of the screening process, potential participants were asked if they had previously been diagnosed with HIV, as this was an exclusion criterion, and they were warned about the potential for false negative self-test results among people on ART. Participants consented to follow-up with short message service (SMS) messages and telephone calls, and also provided consent to extract information related to study outcomes from clinical and laboratory records. A paper-based study register was used to record self-reported HIV status, age, sex and contact details. Participants were required to provide a mobile phone number that was registered in an automated database on enrolment (Table 1). Participants were asked to report their HIVST result by study pre-paid SMS or by returning to the facility or when telephoned as part of follow-up. HIVST results reported by SMS were automatically captured in an electronic database. As all participants provided a mobile number at enrolment, this number could be linked to HIVST results reported by SMS. These results were instantly acknowledged by an automated SMS response (Table 1). Study staff were also sent an SMS notification (mobile phone number and result reported) when a participant sent in a result so that they could transcribe the reported self-test result into the paper-based study register and initiate follow-up procedures for any positive results immediately.

**Table 1:** SMS responses from automated database.

Immediate response to registration of participant mobile number on automated database: *“Welcome to the self-testing pilot*! *Thank you for signing up and we hope to hear from you soon”*.
Response to HIVST self-reported result: 1. Participants with a negative HIVST result (one line across the test strip) were instructed to text “ht 1” by SMS. They were sent an automated response stating: *“Your result is fine but remember this test is not 100% accurate*. *Please return to the Hub/clinic as soon as possible to confirm this result*. *The wellness hub/clinic also provides TB screening and free condoms*!*”*. 2. Participants with a positive HIVST result (two lines across the test strip) were instructed to text “ht 2” by SMS. They were sent an automated response stating: *“Based on your message*, *you need to return to the clinic as soon as possible”*. 3. Participants were instructed to text “ht 0” if they were unsure of the HIVST result. They were sent an automated response stating: *“Your result is not clear*. *It is important that you return for a repeat”*.
Reminder messages were sent once per day for up to 7 days for those who had not reported their HIVST result: *“Are you OK*? *Please reply 'ht 1' if there's 1 line*, *'ht 2' if there's 2 lines or 'ht 0' if you're unsure (free to send) or return to the Wellness Hub or clinic”*. Reminder messages were stopped once the participant reported their HIVST result.
Reminder messages were sent once per week for up to 3 weeks for those who had self-reported a result but had not yet returned for confirmation: *“Looking forward to seeing you at the Wellness Hub or clinic as soon as possible*. *Hub hours are from 9am-1pm and 3pm-7pm*, *Monday-Friday or clinic Hours 8am-4pm*, *Monday-Friday”*.

Participants were sent once daily automated SMS reminders to report their HIVST result for up to 7 days. Messages intentionally did not mention HIV, in order to protect participant confidentiality. Study staff phoned participants who had not reported their HIVST result up to three times, approximately 7, 14 and 21 days after enrolment. Reminder messages stopped once the participant reported their test result, but once weekly messages continued for 3 weeks, encouraging them to return for confirmation. Study staff did telephonic follow-up of all participants who reported positive results by SMS to arrange confirmatory HIV testing. Participants who returned to the clinic received confirmatory testing using rapid finger-prick tests, following the standard national HIV testing algorithm (S1 Fig). Participants with a positive confirmatory test were referred for HIV care (at the same clinic for facility 1 and to a nearby clinic for facility 2 because it had no HIV ART clinic).

The counsellor provided an individual counselling session with content similar to that provided during standard HTS pre-counselling; demonstrated how to use the HIV self-test before the issue of one self-test to use in private; provided simple written and pictorial instructions in Xhosa (the local language) and English, based on manufacturer’s instructions; and gave participants instructions on reporting and follow-up procedures. This process took about 20 minutes, but varied according to individual participant’s needs.

Participants were given a WHO Prequalified OraQuick Rapid HIV-1/2 Antibody Test (OraSure Technologies), an oral fluid test, to use for HIVST. The study materials were dispensed in brown paper packets to assure confidentiality.

### Data collection and outcomes

HIVST results, date of reporting, and the dates of follow-up telephone calls were recorded in the paper-based study register. To preserve confidentiality, the study registers were kept in a locked filing cabinet when not in use and were only accessible to authorised study staff.

Follow-up for eight weeks occurred to determine linkage to services for HIV testing outcomes (reporting HIVST result and confirmatory testing). Participants with newly-diagnosed HIV infection were followed up for six months to assess linkage to ART care.

All information from the study registers was entered into a database. This information was merged with the SMS database to create the study database used in the analysis. Participants with a positive HIVST result were classified as having HIV infection if confirmatory HIV testing was done within eight weeks of enrolment.

Additional information sources, including clinical and laboratory databases, were searched for evidence of HIV status prior to or after study enrolment. The National Health Laboratory Service (NHLS) database, which contains results from all Department of Health clinics in South Africa, was searched for CD4 and HIV viral load results, using name, date of birth, and sex for matching. This was done for all participants regardless of reported result of self-testing.

A CD4 result was used as a marker for confirmed HIV infection and a viral load result was used as a marker for being on ART. Searches of the electronic databases were extended at least five years prior to enrolment, and the effective follow-up period of database search was extended to a minimum of 12 months after enrolment.

To assess linkage to care following HIVST, study staff searched the clinical records at the study facilities for confirmatory HIV test results and the ART initiation date among participants with confirmed HIV infection. Two regional clinical databases (Western Cape Department of Health and the Cape Town City Health Department) were searched, limiting searches to clinics in Khayelitsha, searching for CD4, viral load and care status before and after the HIVST.

### Data analysis

In the analysis, HIV test results reported more than eight weeks after enrolment were treated as “not reported”, and participants who had confirmatory testing more than eight weeks after enrolment were treated as having an unconfirmed HIV outcome. Participants were categorised as having “previously-diagnosed HIV infection” if any clinical or laboratory records provided evidence that the HIV diagnosis was made prior to enrolment. Participants were categorised as “newly diagnosed HIV infection” if record searches did not provide any evidence of a previous diagnosis of HIV, and if confirmatory testing was done within eight weeks of enrolment.

In order to check linkage and remaining in care, we did subgroup analyses of viral load testing, and viral load suppression, in line with the UNAIDS 90-90-90 objectives. PLHIV diagnosed prior to enrolment were classified as being “in care” before enrolment if any clinical or laboratory records provided documentation that they had been in care (defined as HIV clinic visits either pre-ART or on ART) at any time during the 12 months preceding enrolment. They were classified as “in care” after enrolment if any clinical or laboratory records provided documentation that they had been in care after enrolment. Participants were classified as having “care status unknown” if clinical and laboratory record searches did not provide any evidence that they had received HIV care during the specified time period.

The data analysis was primarily descriptive, with results being reported in terms of frequencies and proportions. Pearson’s chi-squared tests were used to assess the statistical significance of differences in participant characteristics according to facility.

## Results

### Recruitment and participant characteristics

Of 714 individuals recorded in the study registers, 639 enrolled and 75 declined.

The reasons given for refusal among the 75 individuals who declined to participate, were that they preferred facility-based HIV testing (n = 19; 25.3%); that they were not ready to have an HIV test (n = 16; 21.3%); they were scared to test (n = 8; 10.7%); they were unwilling to self-test (n = 8; 10.7%); or other reasons (n = 10; 13.3%). The last 14 (18.7%) did not give a reason for refusal.

Of the 639 participants, 453 (70.9%) were recruited at Facility 1, and 186 (29.1%) were recruited at Facility 2. Participant characteristics are shown by facility in Table 2.

**Table 2:** Participant characteristics (N = 639).

Characteristic	Facility 1 (n = 453) n (%)	Facility 2 (n = 186) n (%)	Both facilities (N = 639) n (%)	Pearson’s chi square P-value
Sex				0.03
Female	424 (93.6)	182 (97.9)	606 (94.8)	
Male	29 (6.4)	4 (2.2)	33 (5.2)	
Age group (years)				<0.001
18–24	70 (15.5)	84 (45.2)	154 (24.1)	
25–29	164 (36.2)	46 (24.7)	210 (32.9)	
30–34	103 (22.7)	32 (17.2)	135 (21.1)	
≥35	116 (25.6)	24 (12.9)	140 (21.9)	
Method of reporting HIVST result				<0.001
SMS	288 (63.6)	78 (41.9)	366 (57.3)	
Phone tracing	41 (9.1)	5 (2.7)	46 (7.2)	
Return to facility	14 (3.1)	2 (1.1)	16 (2.5)	
Did not report	110 (24.2)	101 (54.3)	211 (33.0)	

Overall, the majority of participants (94.8%) were female. Compared to Facility 1, Facility 2 had significantly fewer male participants, significantly more participants aged under 25 years and significantly more participants who did not report their HIV result (Table 2).

### Reporting of HIVST results

Of the 639 participants, 428 reported a result: 401 (62.8%) reported a negative HIVST result, 27 (4.2%) reported a positive HIVST result, and 211 (33.0%) did not report their HIVST result. There were an additional 42 HIVST results (40 negative and 2 positive) reported from mobile phone numbers that were not registered in the database. These reports could not be linked to a participant, nor assigned to a reporting category, as it could not be established whether the participants making the reports were subsequently contacted by telephone and reported their HIVST result.

SMS was the most frequent mode of reporting HIVST results. Of the 428 participants who reported an HIVST result, 366 (85.5%) reported by SMS, 46 (10.7%) reported their result when contacted by telephone, and 16 (3.7%) reported their result on returning to the facility. Median time to SMS reporting was one day after enrolment, with 33.9% reporting their result on the day of enrolment, and 75% reporting their result within 5 days of enrolment.

### HIV outcomes (Fig 1)

Laboratory and clinical data search identified additional PLHIV among study participants and this data was integrated with the study data. Of the 639 participants, 57 (8.9%) were confirmed to have HIV infection, with 39 (6.1%) having had HIV infection diagnosed prior to enrolment (previous diagnoses), and 18 (2.8%) being diagnosed after enrolment (new diagnoses). An additional 73 participants (11.4%) were confirmed to be HIV negative (i.e. had a second confirmatory test), and the HIV status of the remaining 509 participants (79.7%) could not be ascertained for study purposes (although according to self-testing guidelines, 330 of these can be assumed negative after one test).

**Fig 1:**
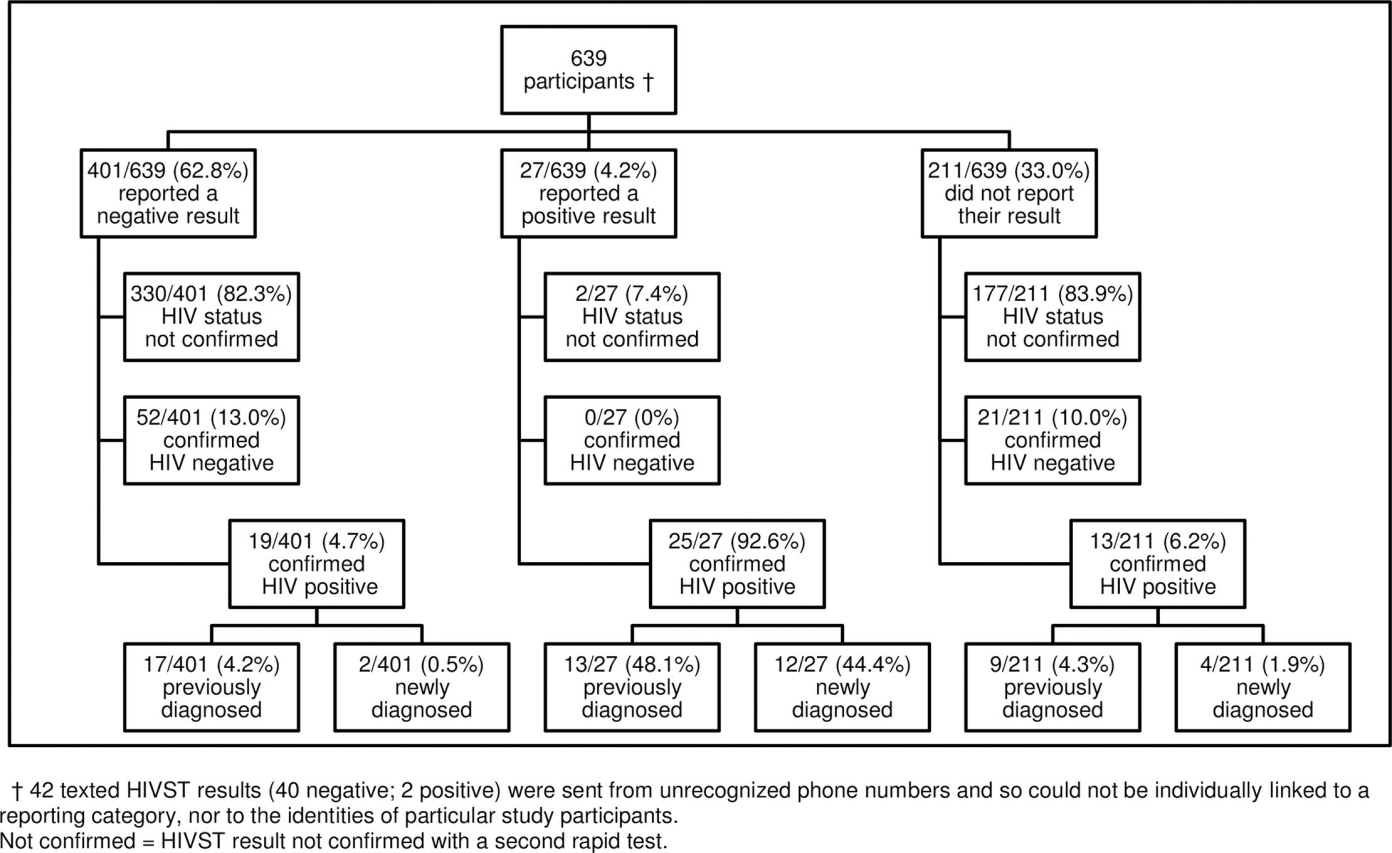
Flow diagram of HIV testing outcomes.

The HIV outcomes are shown according to HIVST reporting category in Fig 1.

Of the 27 participants who reported a positive result (see middle column, Fig 1), 25/27 (92.6%) were confirmed to have HIV infection, with 13/27 (48.1%) being previous diagnoses, and 12/27 (44.4%) being new diagnoses. The HIV status of the remaining two participants could not be determined because searches of clinical records, and the clinical and laboratory databases, did not yield a record of either of them having confirmatory testing following HIVST, or a previous diagnosis of HIV infection.

Of the 401 participants who reported a negative HIVST result (left-hand column, Fig 1), 52 (13.0%) were confirmed to be HIV negative by a second test, and 19 (4.7%) were confirmed to have HIV infection according to data search, with 17 (4.2%) being previous diagnoses, and two (0.5%) being new diagnoses. The HIV status of the remaining 330 (82.3%) could not be confirmed.

Of the 211 participants who did not report their HIVST result (right-hand column, Fig 1), searches of clinical and laboratory records revealed the HIV status of 34 (16.1%); 21 (10.0%) were confirmed to be HIV negative, nine (4.3%) were previous HIV diagnoses, and four (1.9%) were new HIV diagnoses.

**Fig 2:**
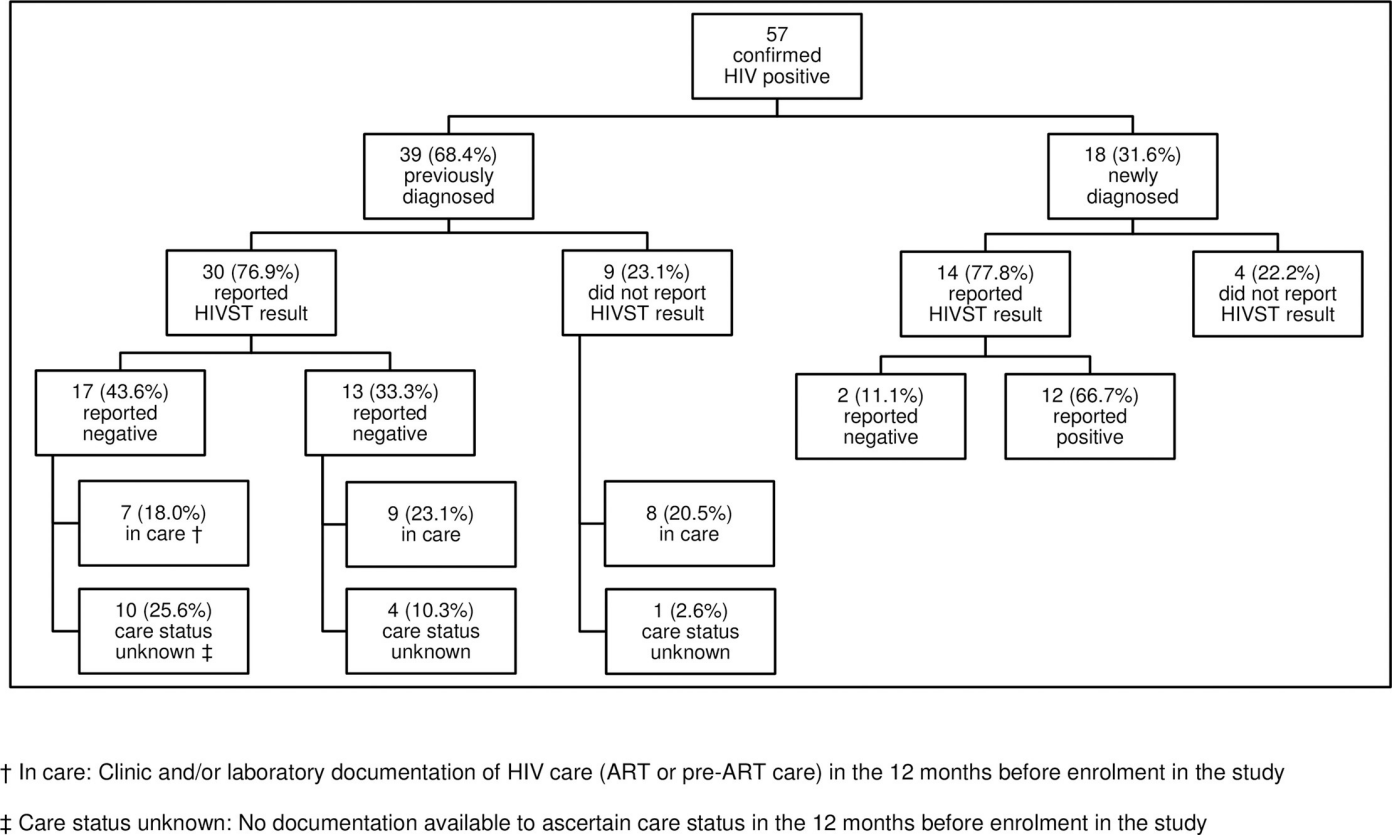
Flow diagram for HIV positive participants.

Fig 2 gives more details of the 57 HIV positive participants.

During the study period, Facility 2 provided services to 6,426 people. Of these, 2067 (32.2%) had standard PICT; 207 (10.0%) people who had declined PICT enrolled in the study, and of these, 134 (6.4%) had documented HIVST outcomes. This suggests a possible 6.4–10.0% increase in HIV testing. Of the 2114 people tested at the facility during the study period, 81 (3.8%) were newly-diagnosed with HIV infection. Nine of these newly-diagnosed infections (11.1%) were initially identified by HIVST and confirmed with standard rapid HIV testing at the facility.

### Care status among participants previously diagnosed as HIV positive

Among the 39 PLHIV with previously-diagnosed HIV infection, 24 (61.5%) were confirmed to be in care before HIVST, of which 23 (59.0%) were confirmed to still be in care after HIVST (Table 3) and one participant (2.6%) did not have their care status confirmed after HIVST. Five (12.8%) with unknown care status before HIVST were confirmed to be in care after HIVST. For 10/39 (25.6%), no documentation was available to indicate their care status before or after HIVST.

**Table 3:** Care status before and after HIVST among participants with previously-diagnosed HIV infection (N = 39).

		After HIVST		Total
		In care	Care status not confirmed	
Care status before HIVST	In care	23 (59.0%)	1 (2.6%)	24 (61.5%)
	Care status not confirmed	5 (12.8%)	10 (25.6%)	15 (38.5%)
		28 (71.8%)	11 (28.2%)	39 (100%)

### ART initiation among participants with newly-diagnosed HIV infection

Of the 18 participants with newly-diagnosed HIV infection, six (33.3%) started ART within six months of HIVST, and additional two (11.1%) started ART more than six months after HIVST. No record was found of the remaining 10 (55.6%) having started ART.

### Continuation of care—Viral load testing and viral suppression

A comparison of viral load testing (indicating in care) and viral suppression (indicating adherence to medication) among PLHIV before HIVST is given in Table 4. Among the 39 participants with previously-diagnosed HIV infection, 14 (35.9%) had a viral load result from the year preceding HIVST and 19 (48.7%) in the year after HIVST (indicating they were in care), of whom 92.9% and 84.2% respectively were virologically suppressed.

**Table 4:** Viral load testing and viral load suppression in the 12 months before and after HIVST among PLHIV who tested with HIVST (N = 39).

		Viral load after HIVST			Total
		<100 copies/ml	≥100 copies/ml	Unknown†	
Viral load before HIVST	<100 copies/ml	9 (23.1%)	2 (5.1%)	2 (5.1%)	13 (33.3%)
	≥100 copies/ml	1 (2.6%)	0 (0%)	0 (0%)	1 (2.6%)
	Unknown†	6 (15.4%)	1 (2.6%)	18 (46.2%)	25 (64.1%)
		16 (41.0%)	3 (7.7%)	20 (51.3%)	39 (100%)

† No viral load result found

Among the 18 participants with newly-diagnosed HIV infection, 5 (27.8%) had a viral load test within a year after HIVST. All five of those tested were virologically suppressed (viral load <100 copies/ml).

## Discussion

This study demonstrated that HIVST was feasible and could increase HIV testing uptake in a setting with high testing coverage, particularly amongst those who declined clinic-based HTS. Despite attempts to exclude people with previously-diagnosed HIV infection, 39 enrolled in the study. The assessment of care status before and after enrolment found that participating in the study did not result in participants disengaging from care, despite some PLHIV with previously-diagnosed HIV infection reporting negative HIVST results.

The availability of an NHLS database containing retrospective CD4 and viral load data allowed important insights into awareness of positive status that participants were not comfortable to reveal at the time of inclusion in the study. Over two thirds of those identified as having confirmed HIV infection had been diagnosed prior to study enrolment, despite efforts to exclude those with known positive status and warning of the possibility of a false negative HIVST if on ART [5,34]. Repeat testing by those on ART has been seen elsewhere [22,35] and suggests that some PLHIV desire to re-confirm their HIV status for themselves, despite their reluctance to disclose their status.

Nearly half of the PLHIV who were previously-diagnosed with HIV infection reported a negative HIVST result. This raises a concern about the possibility of false-negative results, particularly among participants with viral suppression [15] or who had initiated ART soon after infection [17]. A possible consequence of a false-negative HIVST result could be disengagement from care. There was no evidence of disengagement from care in the event of false-negative results in this study. Viral load analysis showed that all but one of participants who were on ART before HIVST, remained in care after HIVST, despite their varied self-reporting to the study. Those with viral load unknown before HIVST may have never been in care or had possibly defaulted from care and were now tentatively engaging again.

Some participants classified as ‘care status unknown’ may have been receiving care from clinics outside Khayelitsha or may have been missed on record searches due to inconsistencies in the spelling of their names or recording their clinical folder numbers.

There is a need for clearer messaging to decrease inappropriate testing (and future HIVST kits will carry a label, reminding users not to use the test if taking ARVs), but our experience suggests it may not be possible to entirely prevent it. The study suggests that there are potential positive outcomes of HIVST for participants with previously-diagnosed HIV infection. Some defaulters from ART returned to treatment and some known HIV-infected people started ART for the first time after using HIVST, suggesting that HIVST enabled re-engagement with care for those who had disengaged, and improved adherence amongst those who were poorly adherent. HIVST may thus facilitate linkage and re-linkage to ART care for PLHIV who may be in denial of their positive HIV status and/or their need for treatment. Further research is required to study reasons for repeat testing (both self-tests and traditional HTS) among PLHIV who are on ART, and its positive and negative impacts, in order to refine messaging and strategies for promotion of testing.

The study started before universal test and treat, so the newly-diagnosed participants with high CD4 levels were not yet eligible to start ART. However, nearly a third had started ART by six months after HIVST, and over half by nine months after HIVST.

Reporting of status by SMS was the preferred method of reporting with participants. Most of those who reported a positive HIVST result returned for confirmatory HIV testing, while few of those who reported a negative result returned for confirmatory testing. This suggests that HIVST can play a triaging role to guide the use of HIV testing services. The relatively high rate of SMS reporting may be attributed to the SMS reporting costs being pre-paid by the researchers, and because participants were informed that they would receive follow-up phone calls if they did not report their result. SMS technology may be a feasible way to report results and facilitate linkage to services although it is limited by frequent changes of mobile numbers in some settings or incorrect reporting because participants misunderstood instructions on how to report their results. This caused possible underestimation of the study HIVST reporting rate.

Applications of SMS messaging in the HIV care field include promoting adherence to ART [36,37], sending appointment reminders to patients [38], and reporting HIV test results to patients [39]. Consideration should be given to potential applications of SMS messaging to enable patients to communicate with providers.

While 39.5% of adults with HIV infection in South Africa are male [4], men were poorly represented in the study. This is largely attributable to the lower attendance at health facilities of men relative to women, and it should be noted that in Khayelitsha, just 34% of those using clinic-based testing in the facilities during the study period were males. However, the particularly low uptake of HIVST by men warrants further investigation. In future, secondary distribution through female participants as well as community and other distribution modalities may increase male HIVST testing. Although this study was limited to participants over 18 years of age (because of legal ethical requirements), adolescents, with high prevalence of HIV infection and relatively low use of health services, are an important group to target by promoting HIVST testing as has been shown in other studies [40].

A major strength of the study was the access to electronic laboratory and clinical data, to corroborate self-reported HIV, to show ART status at inclusion, and to show testing outcomes. The variation in implementation of study procedures at the two sites represents a limitation affecting our ability to interpret uptake in particular.

In conclusion, the study showed that among a predominantly female, clinic-based population with high testing coverage, unsupervised HIVST was a useful addition to HTS and helped to identify new HIV cases. Outcomes indicate that retesting by people already on ART did not result in them disengaging from care and in fact led to some re-engaging with care after self-testing. Further studies will assess the effectiveness of different HIVST distribution models (through community outreach and private pharmacies) to reach those most in need, including males, who can benefit from HIVST as part of HTS.

## Supporting information

S1 Fig South African National HIV testing algorithm. (PDF)

S1 File Data for all participants. Data in Excel.
(XLSX)

S2 File Data for Fig 1. Data in Excel.
(XLSX)

S3 File Data for HIV infected participants. Data in Excel.
(XLSX)

S4 File Data for Fig 1. Data in CSV.
(CSV)
