# Supplementary material for: Investigating the addition of oral HIV self-tests among populations with high testing coverage – Do they add value? Lessons from a study in Khayelitsha, South Africa
Source: PLoS One. 2019 May 2;14(5):e0215454. doi: 10.1371/journal.pone.0215454 (PMC6497254; doi:10.1371/journal.pone.0215454)
Supplement: S1 Fig — (PDF) [file pone.0215454.s001.pdf]

South African National HIV Testing Algorithm

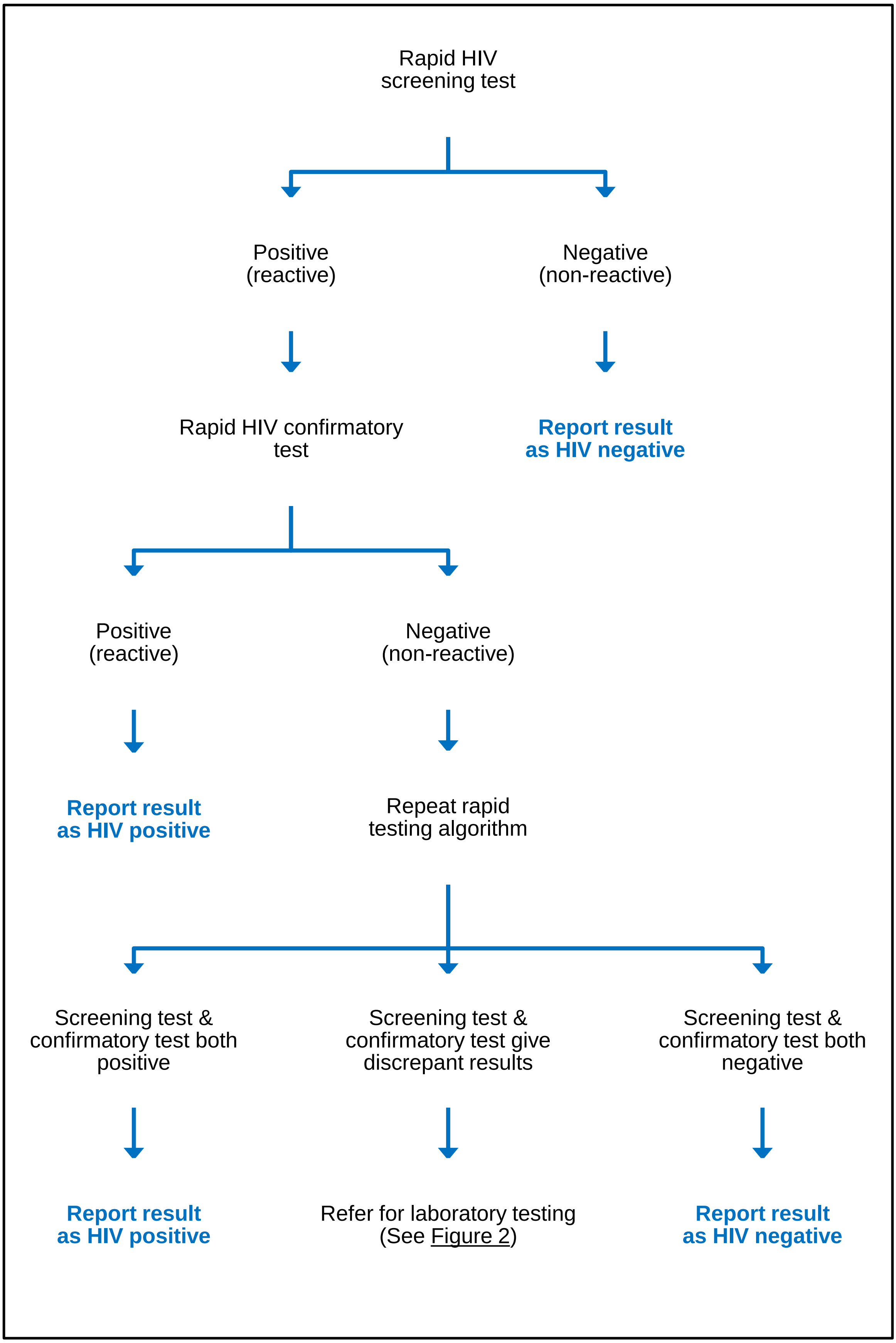

Figure 1: National HIV testing algorithm

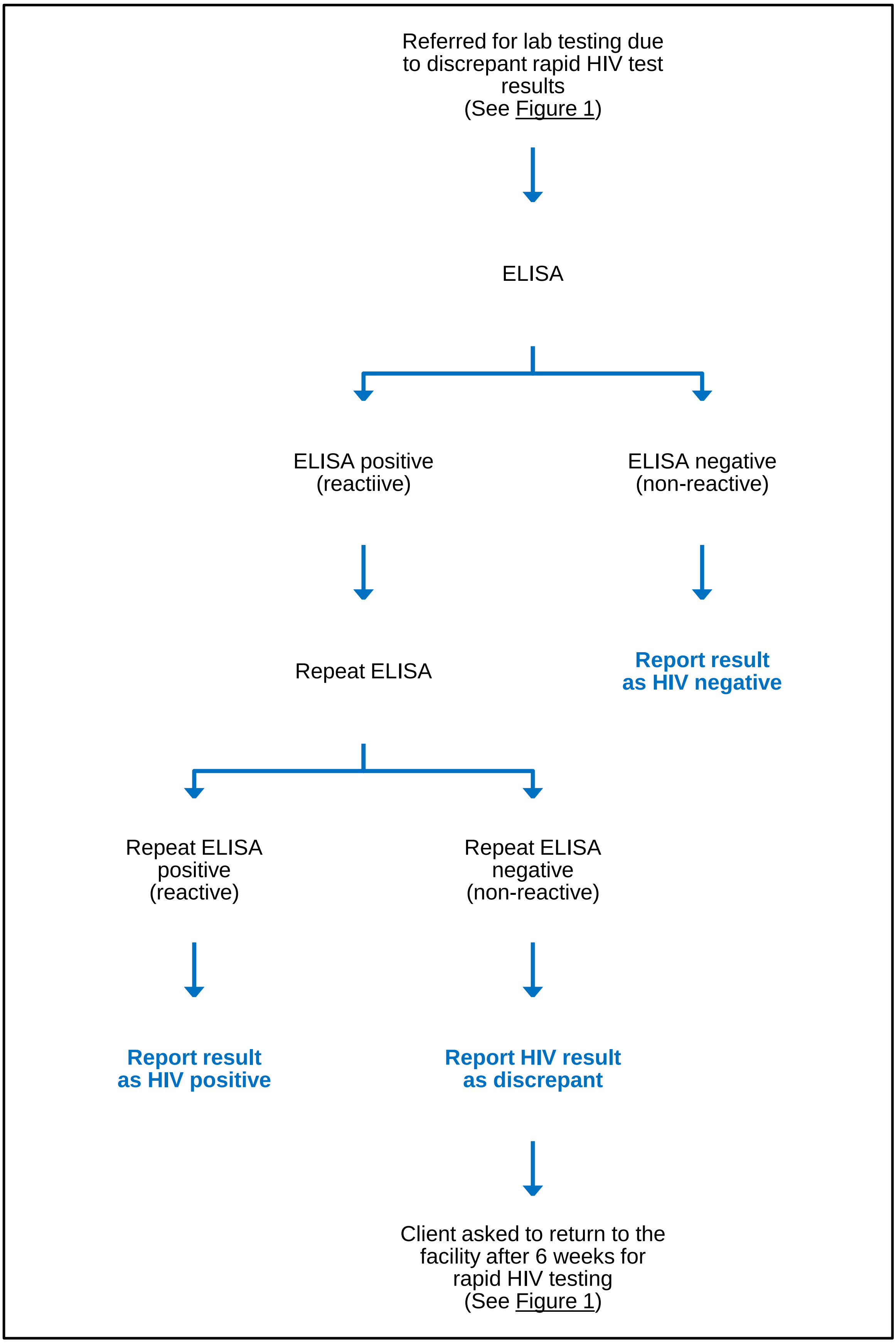

Figure 2: National HIV laboratory testing algorithm to resolve discrepant rapid HIV test results
